# Facile Synthesis of Lacunary Keggin-Type Phosphotungstates-Decorated g-C_3_N_4_ Nanosheets for Enhancing Photocatalytic H_2_ Generation

**DOI:** 10.3390/polym12091961

**Published:** 2020-08-29

**Authors:** Na Lu, Menghan Sun, Xiaoming Wei, Peng Zhang, Zhenyi Zhang

**Affiliations:** 1 Key Laboratory of New Energy and Rare Earth Resource Utilization of State Ethnic Affairs Commission, Key Laboratory of Photosensitive Materials & Devices of Liaoning Province, School of Physics and Materials Engineering, Dalian Minzu University, 18 Liaohe West Road, Dalian 116600, China; luna@dlnu.edu.cn (N.L.); 18241113494@163.com (M.S.); 2 School of Materials Science and Engineering, Zhengzhou University, Zhengzhou 450001, China; zhangp@zzu.edu.cn

**Keywords:** photocatalysis, g-C_3_N_4_, H_2_ generation, Z-scheme mechanism, electron transfer

## Abstract

In this work, the lacunary Keggin-type phosphotungstates of [PW_9_O_34_]^9-^ (PW_9_) clusters were loaded onto the g-C_3_N_4_ nanosheets (NSs) to synthesize the phosphotungstate clusters-decorated 2D heterojunction photocatalysts by using the electrostatic-force driven self-assembly process. The surface charge polarity of g-C_3_N_4_ NSs was changed from a negative to a positive charge through the acidizing treatment. The positively-charged g-C_3_N_4_ NSs allowed the negatively-charged PW_9_ clusters to be adsorbed and deposited onto the g-C_3_N_4_ NSs, forming the PW_9_/g-C_3_N_4_ heterojunction NSs. The as-synthesized samples were characterized by scanning electron microscopy (SEM), X-ray diffraction (XRD), Fourier-transform infrared (FTIR) spectroscopy, and UV-VIS absorption spectra, respectively. The photocatalytic activity tests indicated that, upon simulated sunlight irradiation, the photocatalytic H_2_-generation rate of PW_9_/g-C_3_N_4_ heterojunction NSs (~23.8 μmol h^−1^) was ~3.3 times higher than that of the pure g-C_3_N_4_ NSs (~7.3 μmol h^−1^). The enhanced photocatalytic activity of PW_9_ cluster-decorated g-C_3_N_4_ NSs could be attributed to the enhanced separation process of the photoinduced charge-carriers, due to the Z-scheme-mediate charge transfer behavior across their hetero-interface.

## 1. Introduction

The continuous burning of unsustainable fossil energy sources, which emits large amounts of harmful gases, has induced a global energy crisis and environmental contamination. The development of green renewable energy sources to replace traditional fossil energy sources has become an urgent issue [1,2]. As a kind of pollution-free sustainable fuel, hydrogen (H_2_) has been regarded as an ideal candidate for the application of energy supply in future society [3,4]. Splitting water through the additional electric or photic energy is a common method to obtain the H_2_. Since the H_2_ generation from the photo-driven water splitting over the TiO_2_ semiconductor photocatalyst was first reported in 1972 [5], this photochemical reaction has been widely used in the research area of solar-to-fuels conversion, mainly owing to its low cost and low energy consumption [6,7,8]. However, because the classical photocatalyst of TiO_2_ nanostructures is a wide bandgap semiconductor (3.0–3.2 eV), it can only absorb the UV light that accounts for 3–5% in the solar spectrum [9,10]. Thus, the development of narrow bandgap semiconductors as the photocatalysts for H_2_ generation has emerged as a hot topic in the area of solar-to-fuels conversion.

As a polymeric semiconductor made of carbon and nitrogen elements, graphite C_3_N_4_ (g-C_3_N_4_) material has attracted increasing interest in the area of photocatalytic H_2_ generation [11,12]. This kind of metal-free semiconductor with a 2D nanosheet structure can open new prospects for the application of solar-to-fuels conversion because it is an abundant, cheap and stable semiconductor, with a suitable energy-level position for reducing protons. However, the fast recombination of the photoinduced charge-carriers often results in poor photocatalytic activity of the g-C_3_N_4_ NSs [13]. To overcome this problem, much effort has been contributed to couple the g-C_3_N_4_ NSs with an appropriate photoactive material to construct the 2D heterojunction photocatalyst toward high-efficient photocatalytic H_2_ generation. 

As a class of polyoxometalates, lacunary Keggin-type phosphotungstates have been extensively investigated as photosensitizers for enhancing the photocatalytic activity of the semiconductor photocatalysts for H_2_O_2_ production, H_2_ generation, CO_2_ reduction, pollution degradation, and organic material synthesis [14,15,16]. Similar to the inorganic semiconductors, the phosphotungstates also have the photoinduced electron transition behavior from the HOMO (Highest Occupied Molecular Orbital) to LUMO (Lowest Unoccupied Molecular Orbital). In general, the charge-transfer from the ligand to metal (O to W) in the phosphotungstates enable them to possess a high light absorption coefficient. Meanwhile, the enriched metal nodes of such W in the phosphotungstates can boost the photoinduced multi-electron redox-processes for fulfilling the various photo-chemical reactions [17]. Furthermore, when the lacunary Keggin-type phosphotungstates are dissolved in a water solution, the negatively-charged phosphotungstates can be obtained due to the ionization effect, thereby leading the phosphotungstates to be easily absorbed onto the substrate with the positive charge [18,19]. Notably, the surface charge polarity of g-C_3_N_4_ NSs is controllable by the acidizing treatment. It is concluded that the lacunary Keggin-type phosphotungstates are one of the ideal guest photosensitizers for coupling with the g-C_3_N_4_ NSs host, through the electrostatic-force driven self-assembly process for enhancing the photocatalytic H_2_ generation of g-C_3_N_4_ NSs. 

In this work, we adjusted the surface charge polarity of g-C_3_N_4_ to positive by using the acidizing treatment, and loaded the lacunary Keggin-type phosphotungstates of [PW_9_O_34_]^9-^ (PW_9_) clusters onto the g-C_3_N_4_ NSs, forming the PW_9_/g-C_3_N_4_ heterojunction NSs, via a facile self-assembly process due to the electrostatic interaction. We expected to utilize the photoinduced electrons on the LUMO of PW_9_ component transfer to the valence band (VB) of g-C_3_N_4_ component for extending the lifetime of photoinduced electrons on the conduction band (CB) of g-C_3_N_4_ for executing protons reduction and improving the photocatalytic activity of the PW_9_/g-C_3_N_4_ heterojunction NSs for H_2_ generation. It is believed that our work will provide a new platform to construct the ultra-small polyoxometalates cluster-decorated g-C_3_N_4_ NSs for highly-efficient photocatalytic solar-to-fuels conversion.

## 2. Experimental

### 2.1. Synthesis of the PW_9_, g-C_3_N_4_ NS, and PW_9_/g-C_3_N_4_ Heterojunction NSs

The lacunary Keggin-type phosphotungstates of Na_9_[A-α-PW_9_O_34_]·7H_2_O (named as PW_9_) were synthesized by using the previously reported method [20]. The g-C_3_N_4_ was synthesized through the traditional thermal polymerization method by using the urea as the precursor [21]. To obtain the PW_9_/g-C_3_N_4_ heterojunction NSs, 100 mg of the g-C_3_N_4_ bulk was ground and then dispersed into deionized water under the ultrasonic treatment for 90 min at room temperature, thereby achieving the g-C_3_N_4_ nanosheets (NSs). Afterward, 1 M of HCl solution was added dropwise into the above g-C_3_N_4_ nanosheets-suspended water solution to adjust the surface charge polarity of the NSs. After that, 100 mg of the PW_9_ was dissolved into the above solution with vigorous stirring for 4 h. The precipitate in the solution was separated by centrifugation treatment. Finally, the obtained mixture was dried at 80 °C for 12 h, which was then under annealing at 300 °C for 2 h in vacuum. Thus, the PW_9_/g-C_3_N_4_ heterojunction NSs were synthesized. 

### 2.2. Characterization

The structure and morphology of the as-synthesized samples were observed by scanning electron microscopy (Field Emission-SEM; S-4800, Hitachi, Tokyo, Japan) and transmission electron microscopy (TEM) (JEM-2100, JEOL, Tokyo, Japan). The X-ray diffraction (XRD) patterns of the as-fabricated samples were studied by X-ray diffractometer (XRD-6000, Shimadzu, Tokyo, Japan) with a Cu Kα line of 0.1541 nm and the radiation is from 10° to 65° at a scanning rate at 2°/min. The Fourier-transform infrared (FTIR) spectra were recorded on Magna 560 FTIR spectrometer (Thermo Nicolet Corporation, Madison, WI, USA) with a resolution of 1 cm^−1^. The UV-VIS absorption spectra of the samples were recorded on a Lambda 750 UV/VIS/NIR spectrophotometer (Perkin Elmer, Waltham, MA, USA). The specific surface areas of the as-prepared samples were measured with a Micromeritics ASAP-2020 instrument (USA), and analyzed by the Brunauer–Emmett–Teller (BET) method. The surface charge polarity of the samples was measured with a dynamic light scattering spectrophotometer and isoelectric point determination with zeta potential analysis (SZ-100, Horiba, Tokyo, Japan).

### 2.3. Photocatalytic H_2_ Generation

Five milligrams of the as-synthesized samples were dispersed into 10 mL of a water solution containing triethanolamine (TEOA, 15 vol.%) and chloroplatinic acid (H_2_PtCl_6_, 10 μL, 12 mM). Then, the mixture solution was sealed in a quartz reactor and then ventilated with argon gas for 10 min to drive away the residual air. Afterward, the reactor was exposed under simulated sunlight (300-W Xe lamp, PLS-SXE300UV, Beijing, China, coupled with an AM 1.5 filter, the wavelength range of light was from 320–2500 nm, the light density was 100 mW cm^−2^, and the photon flux of the lamp was 0.17 μmol s^−1^ m^−2^). The produced gas was periodically analyzed by a gas chromatograph (GC) equipped with a thermal conductivity detector (TCD) (Beifen-Ruili Analytical Instrument, SP-3420A, Beijing, China).

## 3. Results and Discussion

**Figure 1:**
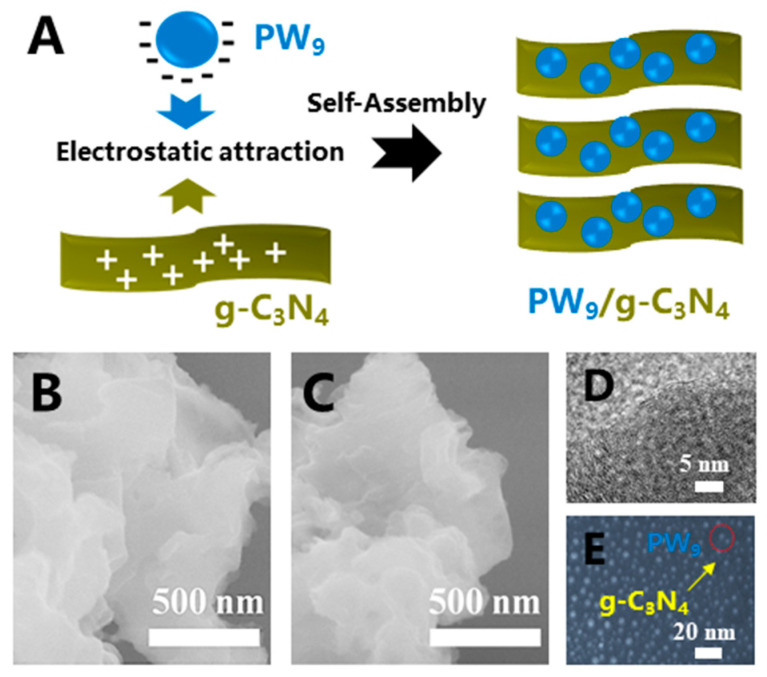
(**A**) Schematic diagram of the self-assembly process of the PW_9_/g-C_3_N_4_ heterojunction NSs, based on the electrostatic force between these two hetero-components; SEM images of (**B**) g-C_3_N_4_ NSs and (**C**) PW_9_/g-C_3_N_4_ heterojunction NSs; the HRTEM image (**D**) and the dark-field STEM image (**E**) of the heterojunction NSs.

To synthesize the PW_9_/g-C_3_N_4_ heterojunction NSs, the g-C_3_N_4_ bulk was crushed into the g-C_3_N_4_ nanosheets (NSs), at first, through the ultrasonic method. Then the obtained g-C_3_N_4_ NSs underwent the acidizing treatment to adjust their surface charge polarity from −21 mV to +6.6 mV. Afterward, the positively-charged g-C_3_N_4_ NSs were suspended into the water solution of PW_9_ to enable the negatively-charged PW_9_ to be self-assembled onto the g-C_3_N_4_ NSs, based on the electrostatic force [22,23]. Finally, the target sample of PW_9_/g-C_3_N_4_ heterojunction NSs was synthesized for further investigation. Figure 1B shows the scanning electron microscopy (SEM) image of the pure g-C_3_N_4_ NSs. It can be seen that the sheet-like g-C_3_N_4_ has a relatively smooth surface. After PW_9_ loading, there was also no change on the surface roughness of the g-C_3_N_4_ NSs in the PW_9_/g-C_3_N_4_ composite NSs (Figure 1C). In order to further investigate the as-prepared PW_9_/g-C_3_N_4_ heterojunction NSs, the high-resolution transmission electron microscopy (HRTEM) and the dark-filed scanning mode TEM were carried out. Although the lattice-fringe spacing of both PW_9_ and g-C_3_N_4_ cannot be observed in HRTEM (Figure 1D), further investigation, by using dark-filed scanning mode TEM (STEM), indicated that the PW_9_ clusters with sizes of 3–5 nm were decorated on the surface of g-C_3_N_4_ NSs in their heterojunction NSs (Figure 1E). In addition, the specific surface areas of PW_9_/g-C_3_N_4_ heterojunction NSs (~68.7 m^2^/g) was similar to that of g-C_3_N_4_ (~53.1 m^2^/g), as shown in Figure S1. 

**Figure 2:**
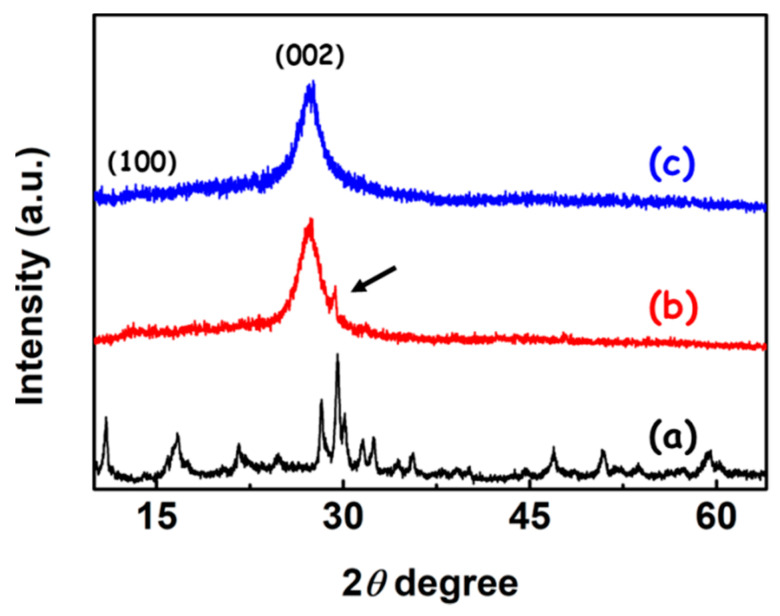
XRD patterns of the as-synthesized samples: (a) PW_9_; (b) PW_9_/g-C_3_N_4_ heterojunction NSs; (c) g-C_3_N_4_ NSs.

The phase structures of the as-synthesized samples were identified through X-ray diffraction (XRD) patterns. As shown in Figure 2, the two intense diffraction peaks around 28°~30° can be observed on the XRD pattern of the PW_9_, which is in accordance with the feature peaks of the PW_9_ reported in the literature [24]. Meanwhile, the other peaks on the diffraction pattern of the Na-PW_9_ were also matched with that of the reported PW_9_, confirming the obtained lacunary Keggin-type phosphotungstates of PW_9_. In the case of g-C_3_N_4_ NSs, the two feature diffraction peaks, belonging to the periodic structure of intra-planar tri-s-triazine and the interlayer stacking of conjugated aromatic structures of g-C_3_N_4_ NSs were found at 13.1° and 27.4°, respectively [25,26,27,28]. This is in agreement with the graphite structure of carbon nitride. When the modification of g-C_3_N_4_ NSs with PW_9_ clusters took place, the feature peaks originated from the Na-PW_9_ with the center at 29.3° and the g-C_3_N_4_ NSs with centers at 13.1° and 27.4° and appeared on the XRD pattern of the formed composite. This result suggests that the g-C_3_N_4_ NSs were decorated with the lacunary Keggin-type phosphotungstates of PW_9_ clusters. 

**Figure 3:**
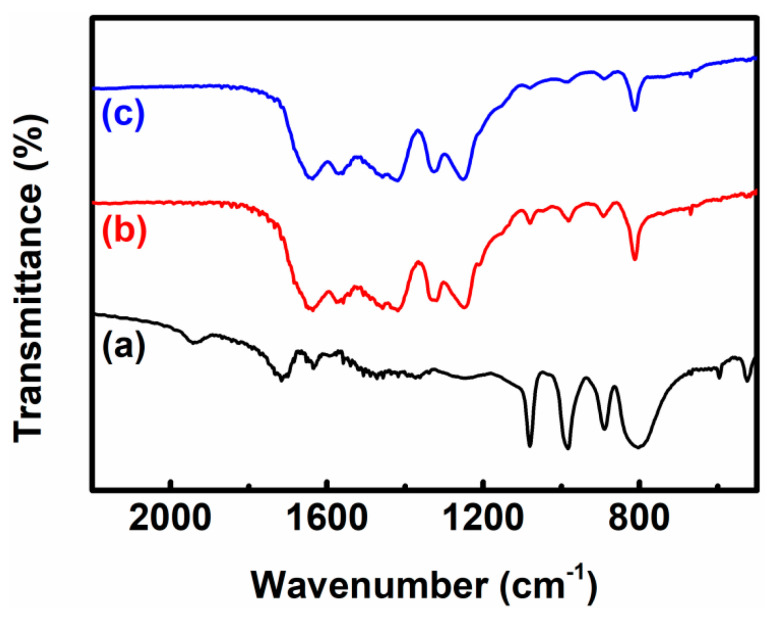
FTIR spectra of the as-synthesized samples: (a) PW_9_; (b) PW_9_/g-C_3_N_4_ heterojunction NSs; (c) g-C_3_N_4_ NSs.

To further confirm the existence of the PW_9_ clusters on the g-C_3_N_4_ NSs, the Fourier-transform infrared (FTIR) spectra of the PW_9_/g-C_3_N_4_ heterojunction NSs along with the corresponding single hetero-components were tested, as shown in Figure 3. For the pure PW_9_, the classical stretching vibrations related to the W-O-W, W-O, and P-O bonds were positioned around the 804/890, 984, and 1077 cm^−1^, respectively [29,30]. It further proves the formation of lacunary Keggin-type phosphotungstates of PW_9_. In the case of pure g-C_3_N_4_ NSs, the vibration bands between 1200 and 1650 cm^−1^ were ascribed to the CN heterocycles [24,31]. The band at 811 cm^−1^ is attributed to the feature vibration mode of s-triazine ring unit [32]. These observations are consistent with the literatures. The feature stretching vibration bands of both PW_9_ and g-C_3_N_4_ could be found on the FTIR spectrum of the PW_9_/g-C_3_N_4_ heterojunction NSs, powerfully confirming the successful decoration of the PW_9_ clusters onto the g-C_3_N_4_ NSs. Notably, as compared with pure g-C_3_N_4_, the feature band at 811 cm^−1^ was slightly shifted to 809 cm^−1^, which might be the cause of the chemical interaction between PW_9_ and g-C_3_N_4_ produced by the electrostatic adsorption process.

**Figure 4:**
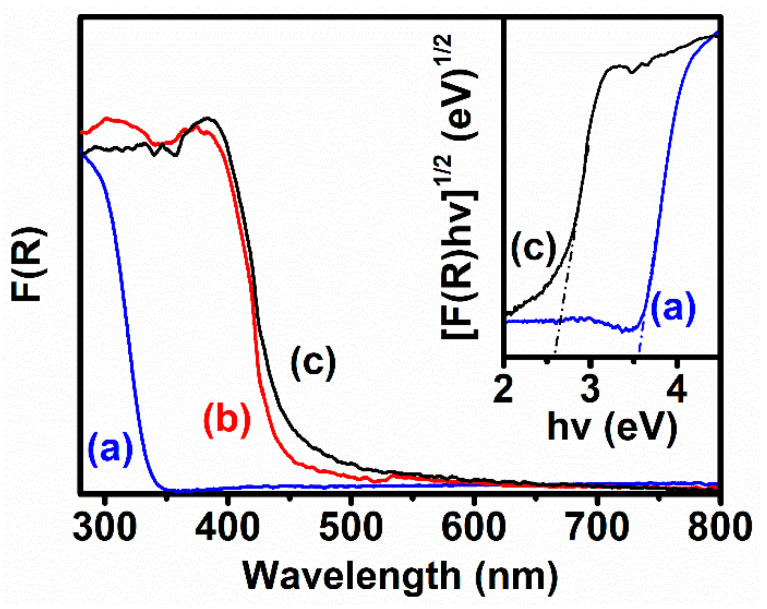
UV-VIS absorption spectra of the as-synthesized samples: (a) PW_9_; (b) PW_9_/g-C_3_N_4_ heterojunction NSs; (c) g-C_3_N_4_ NSs; the inset were plots of the [F(R)(hυ)]^1/2^ versus hυ of (a) PW_9_; (c) g-C_3_N_4_ NSs.

The light absorption behaviors of the as-synthesized samples were evaluated through UV-VIS absorption spectra. As observed in Figure 4, the absorption edge of the pure PW_9_ is located around 350 nm, corresponding to the forbidden gap of ~3.5 eV between HOMO and LUMO of the PW_9_ [15,33]. Meanwhile, the absorption edge of pure g-C_3_N_4_ NSs appears at ~480 nm, suggesting the ~2.6 eV of the bandgap of the g-C_3_N_4_ NSs [34]. The above results indicate that the PW_9_ and g-C_3_N_4_ NSs are the UV and visible absorbers, respectively, during the photo-excitation process. After loading the PW_9_ clusters onto the surface of g-C_3_N_4_ NSs, the formed PW_9_/g-C_3_N_4_ heterojunction NSs displayed the blue-shift behavior of the absorption edge, due to the introduction of the UV-light-active PW_9_ hetero-component. By combining the results of the SEM, TEM, XRD, FTIR, and UV-VIS absorption spectra, we confirm that the PW_9_/g-C_3_N_4_ heterojunction NSs with a well-distribution of PW_9_ clusters were successfully synthesized by using the self-assembly process driven by the electrostatic force. 

**Figure 5:**
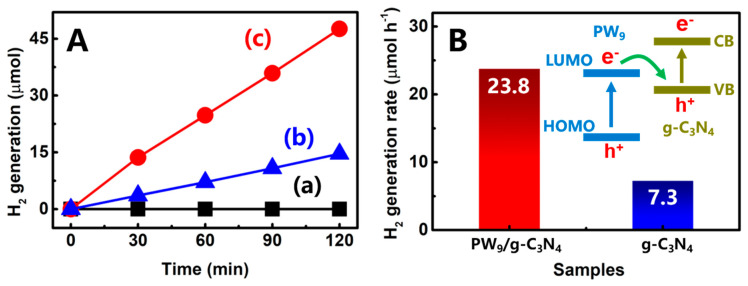
(**A**) Time-dependent photocatalytic H_2_-gereration plots over the different samples under simulated sunlight irradiation: (a) PW_9_; (b) g-C_3_N_4_ NSs; (c) PW_9_/g-C_3_N_4_ heterojunction NSs; (**B**) H_2_-generation rates of the PW_9_/g-C_3_N_4_ heterojunction and g-C_3_N_4_ NSs.

Photocatalytic activity of the as-synthesized PW_9_/g-C_3_N_4_ heterojunction NSs for H_2_ generation was assessed under simulated sunlight excitation in the presence of the sacrificial agent of triethanolamine (TEOA) to quench the photoinduced hole. As observed in Figure 5A, there is no observable H_2_ generation under simulated sunlight irradiation for 2 h, when using the single PW_9_ as the photocatalyst. Meanwhile, the pure g-C_3_N_4_ NSs only displayed a poor photocatalytic activity for H_2_ generation (~14.6 μmol in 2 h-irradiation). After self-assembling the PW_9_ clusters onto the g-C_3_N_4_ NSs to form the PW_9_/g-C_3_N_4_ heterojunction NSs, the photocatalytic activity of the heterojunction NSs for H_2_ generation remarkably increased to ~47.6 μmol in 2 h-irradiation. The H_2_ generation rate of the PW_9_/g-C_3_N_4_ heterojunction NSs was 3.3 times higher than that of the g-C_3_N_4_ NSs, as summarized in Figure 5B. Notably, this photocatalytic activity is almost the highest one among the values reported for other g-C_3_N_4_-based photocatalytic systems (Table 1). Moreover, the solar-to-hydrogen (STH) efficiency has become an important index for evaluating the photocatalytic activity of hydrogen production. The STH efficiency under simulated sunlight irradiation can be calculated according to the following equation [35,36]:

(1) $$\mathrm{STH}=\frac{\mathrm{Energy}\;\mathrm{of}\;\mathrm{generation}\;\mathrm{of}\;\mathrm{hydrogen}\;\mathrm{by}\;\mathrm{water}\;\mathrm{splitting}}{\mathrm{Solar}\;\mathrm{energy}\;\mathrm{irradiating}\;\mathrm{the}\;\mathrm{reaction}\;\mathrm{cell}}\times 100\%$$

After calculating, the STH of PW_9_/g-C_3_N_4_ heterojunction NSs was determined to be about 0.26% at room temperature.

It should be pointed out that the LUMO potential of PW_9_ does not satisfy the demand of proton reduction, while the g-C_3_N_4_ possesses a suitable conduction band (CB) potential for fulfilling the H_2_ generation [37,38]. Thus, we could deduce that the photocatalytic sites in the PW_9_/g-C_3_N_4_ heterojunction NSs should be located at the surface of g-C_3_N_4_ hetero-component [39,40]. Meanwhile, the PW_9_ hetero-component serves as the photosensitizer to boost the separation process of the photoinduced charge-carriers of the g-C_3_N_4_ hetero-component. According to the energy band structures of the reported Keggin-type phosphotungstates and g-C_3_N_4_ NSs, the LUMO position is a little higher than the valence band (VB) position of the g-C_3_N_4_ NSs, as illustrated in the inset of Figure 5B. Thus, when the PW_9_/g-C_3_N_4_ heterojunction NSs is excited by simulated sunlight that contains the photon energies in both UV and visible light regions, the photoinduced electron-hole pairs are generated on the LUMO and VB of the PW_9_ and g-C_3_N_4_ hetero-components, respectively [41,42]. Owing to the potential difference between the LUMO of PW_9_ and the VB of g-C_3_N_4_, the photoinduced electrons on the LUMO of PW_9_ could transfer to the VB of g-C_3_N_4_ in the heterojunction NSs, thereby extending the lifetimes of the photoinduced electrons on the CB of g-C_3_N_4_ for implementing the photocatalytic H_2_ generation [43]. In this way, the Z-scheme photocatalytic mechanism can be employed to explain the enhanced photocatalytic activity of the PW_9_/g-C_3_N_4_ heterojunction system.

**Figure 6:**
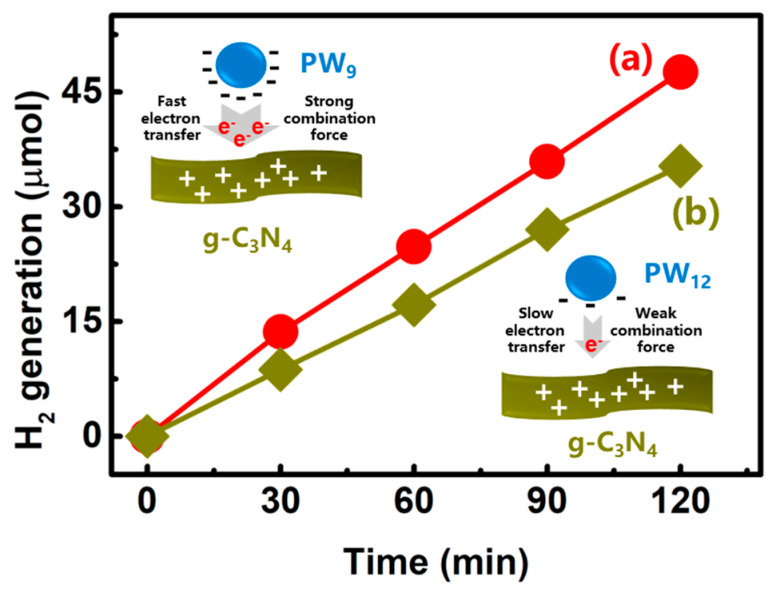
Time-dependent photocatalytic H_2_-generation plots over (a) PW_9_/g-C_3_N_4_ heterojunction NSs and (b) PW_12_/g-C_3_N_4_ heterojunction NSs upon simulated sunlight irradiation. The insets showing the interfacial adsorption and electron transfer abilities of the PW_9_/g-C_3_N_4_ heterojunction and PW_12_/g-C_3_N_4_ heterojunction NSs.

If the Z-scheme electron-transfer process is the main factor for enhancing the photocatalytic activity of PW_9_/g-C_3_N_4_ heterojunction NSs, the hetero-interface combination force between these two hetero-components should have an influence on the photocatalytic H_2_ generation of the NSs. In order to investigate this hypothesis, we synthesized another heterojunction NSs of g-C_3_N_4_, decorated with the normal Keggin-type phosphotungstic acid (H_3_PW_12_O_40_) (PW_12_). Because the number of the negative charges in PW_12_ is less than that in PW_9_ when dissolving them in water solution, the combination force between the PW_12_ and g-C_3_N_4_ should be lower than that in the PW_9_ and g-C_3_N_4_ system. In theory, a strong combination force between the hetero-components would build a high-quality of the transport channel for electron transfer between the hetero-components [51,52]. Thus, the interfacial electron transfer process in the PW_9_/g-C_3_N_4_ heterojunction NSs should be more effective for driving the Z-scheme photocatalytic H_2_ generation as compared to the PW_12_/g-C_3_N_4_ heterojunction NSs (the insets of Figure 6) [53]. The comparison study of the photocatalytic activities of the above two heterojunction NSs indicated that the H_2_-generation activity of PW_9_/g-C_3_N_4_ heterojunction NSs (~47.6 μmol in 2 h irradiation) was ~1.3 times higher than that of the PW_12_/g-C_3_N_4_ heterojunction NSs (~35.3 μmol in 2 h irradiation). This evidence further confirms a fast Z-scheme charge-transfer process occurred in the intimate hetero-interface of PW_9_/g-C_3_N_4_ for enhancing the photocatalytic H_2_ generation.

## 4. Conclusions

In summary, the lacunary Keggin-type phosphotungstates/g-C_3_N_4_ heterojunction NSs were synthesized through self-assembling the PW_9_ clusters onto the g-C_3_N_4_ NSs, based on the electrostatic adsorption process. When adjusting the surface charge polarity of g-C_3_N_4_ NSs from the negative to the positive charge in the PW_9_-dissolved solution, it was found that the intimate hetero-interface between PW_9_/g-C_3_N_4_ heterojunction NSs could be built for boosting the Z-scheme charge-transfer process. Upon simulated sunlight irradiation, the lifetimes of the photoinduced electrons on the g-C_3_N_4_ hetero-component were prolonged in the heterojunction NSs. Thus, a ~3.3-fold enhancement of the photocatalytic activity for H_2_ generation was observed over the PW_9_/g-C_3_N_4_ heterojunction NSs as compared to the pure g-C_3_N_4_ NSs. Our work will provide a new platform to construct the ultra-small sized polyoxometalates clusters-based Z-scheme photocatalysts for enhancing the photocatalytic solar-to-fuels conversion.

## Figures and Tables

**Table 1:** Photocatalytic activity of reported g-C_3_N_4_-based photocatalysts for H_2_ evolution.

Catalyst	Precursor	Light Source	Activity	Ref.
Ag/g-C_3_N_4_/TiO_2_	Melamine	300 W Xe lamp with AM 1.5 filter	1.5 μmol/h	[44]
W_18_O_49_/g-C_3_N_4_	Urea	300 W Xe lamp with λ > 420 nm filter	3.69 μmol/h	[45]
g-C_3_N_4_/MnO_2_	Urea	300 W Xe lamp with λ > 420 nm filter	5.53 μmol/h	[46]
ZnIn_2_S_4_/g-C_3_N_4_	Melamine	300 W Xe lamp with λ > 420 nm filter	14.1 μmol/h	[47]
W_18_O_49_/g-C_3_N_4_	Melamine	300 W Xe lamp with λ > 420 nm filter	18.25 μmol/h	[48]
Zn-AgIn_5_S_8_/g-C_3_N_4_	Urea	300 W Xe lamp with λ > 420 nm filter	17.32 μmol/h	[49]
MoS_2_/g-C_3_N_4_	Urea	300 W Xe lamp with λ > 420 nm filter	19.66 μmol/h	[50]
PW_9_/g-C_3_N_4_	Urea	300 W Xe lamp with AM 1.5 filter	23.8 μmol/h	This work

## Supplementary Materials

The following are available online at https://www.mdpi.com/2073-4360/12/9/1961/s1, Figure S1: Nitrogen adsorption-desorption isotherms of the as-synthesized samples: (a) g-C_3_N_4_, (b) PW_9_/g-C_3_N_4_ heterojunction NSs.
