# Supplementary material for: Facile Synthesis of Lacunary Keggin-Type Phosphotungstates-Decorated g-C3N4 Nanosheets for Enhancing Photocatalytic H2 Generation
Source: Polymers (Basel). 2020 Aug 29;12(9):1961. doi: 10.3390/polym12091961 (PMC7564915; doi:10.3390/polym12091961)
Supplement: Supplementary file 1 [file polymers-12-01961-s001.pdf]

Supplementary Material

Facile Synthesis of Lacunary Keggin-Type Phosphotungstates-Decorated g-C_3N_4 Nanosheets for Enhancing Photocatalytic H_2 Generation

Lu Na [1], Menghan Sun [1], Xiaoming Wei [1,*], Peng Zhang [2] and Zhenyi Zhang [1,*]

[1] Key Laboratory of New Energy and Rare Earth Resource Utilization of State Ethnic Affairs Commission, Key Laboratory of Photosensitive Materials & Devices of Liaoning Province, School of Physics and Materials Engineering, Dalian Minzu University, 18 Liaohe West Road, Dalian 116600, China; luna@dlnu.edu.cn (L.N.); 18241113494@163.com (M.S.)

[2] School of Materials Science and Engineering, Zhengzhou University, Zhengzhou 450001, China; zhangp@zzu.edu.cn

[*] Correspondence: xmwei@dlnu.edu.cn (X.W.); zhangzy@dlnu.edu.cn (Z.Z.)

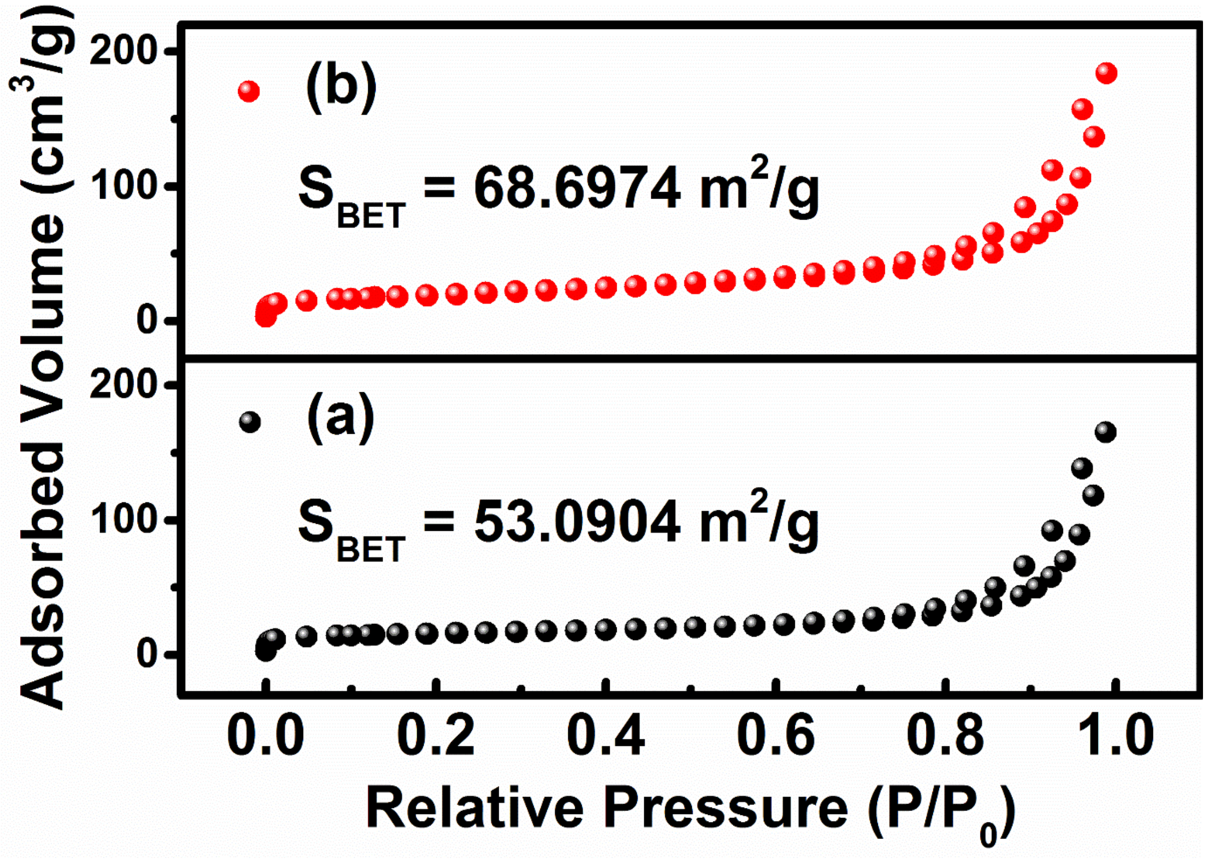

Figure S1. Nitrogen adsorption-desorption isotherms of the as-synthesized samples: (a) g-C_3N_4, (b) PW_9/g-C_3N_4 heterojunction NSs.
